# Health-related quality of life and the ability to perform activities of daily living: a cross-sectional study on 1079 war veterans with ankle-foot disorders

**DOI:** 10.1186/s40779-017-0146-1

**Published:** 2017-11-29

**Authors:** Mostafa Allami, Amir Yavari, Amir Karimi, Mehdi Masoumi, Mohammadreza Soroush, Elahe Faraji

**Affiliations:** Janbazan Medical and Engineering Research Center (JMERC), NO.17, Farrokh St., Moghaddas Ardebily Ave., Chamran Highway, Tehran, Iran

**Keywords:** Activities of daily living, Instrumental activities of daily living, Quality of life, Ankle-foot injuries

## Abstract

**Background:**

The ankle-foot injuries are among the war-related injuries that cause many serious secondary problems for a lifetime. This nationwide study aimed to assess health-related quality of life and the ability to perform activities of daily living in veterans with ankle-foot injuries due to the Iran-Iraq war.

**Methods:**

A total of 1079 veterans with ankle-foot injuries were enrolled in a cross-sectional study from 2014 to 2016. Demographic characteristics, including age, gender, marital status, disability percent, educational level, employment and additional injuries, were collected. The ability to perform daily activities was assessed using the Barthel activities of daily living (ADL) and Lawton instrumental activities of daily living (IADL) Indexes. Physical and mental health-related quality of life (HRQOL) data were measured via the SF-36 subscales. The data were compared with those of bilateral lower limb amputees (BLLAs) and of the general Iranian population. Statistical analyses, including Pearson’s correlation coefficient, one-sample t-test and analysis of variance (ANOVA), were performed using SPSS16.0. A multiple linear regression model was used to determine the contribution of independent variables to the Physical Component Summary (PCS) and Mental Component Summary (MCS).

**Results:**

The highest and lowest scores were observed for mental health (48.93 ± 20.69) and bodily pain (28.16 ± 21.74), respectively. The mean scores of veterans with ankle-foot injuries on the SF-36 were significantly lower on all eight measures than those of the general Iranian male population and of the bilateral lower limb amputees (*P* < 0.001). The mean scores of ADLs and IADLs were 83.9 ± 16.3 and 5.3 ± 2.0, respectively. The higher dependency in ADLs (*P* < 0.001) and IADLs (P < 0.001), the higher disability rate (P < 0.001) and additional injury (P < 0.001) were significant determinants of the PCS. ADL (P < 0.001) and IADL (P < 0.001) limitations, additional injury (P < 0.001), history of hospitalization in the year preceding the study (*P* = 0.007) and employment (*P* = 0.001) were reported as determinants of the MCS.

**Conclusion:**

The results strongly suggest that veterans with ankle-foot injuries suffer from critically poor health-related quality of life. The main predicting factors of HRQOL were the disability to perform ADLs/IADLs, suffering two or more injuries, a history of hospitalization in the year preceding the study and unemployment.

## Background

The longest conventional warfare of the twentieth century occurred between Iran and Iraq (1980–1988). This conflict left more than 550,000 injured veterans, many of whom have been suffering from related disabilities for nearly three decades. According to the data obtained from the Veterans and Martyrs Affair Foundation (VMAF), Tehran, Iran, the total number of veterans that left the eight-year war with ankle-foot injuries was 10,227 [1]. If they do not use walking aids or if they are left untreated, these individuals will show the secondary effects of these injuries mainly due to overuse of the contralateral ankle-foot [2]. Most of these secondary effects result in a limited range of motion in joints, neurological damage, chronic pain and discrepancy in the lower limbs. All of these problems cause mobility and transfer difficulties in both personal and social affairs, resulting in dependence on family members and others [3].

Due to the extended lapse in time since the end of the war, the majority of veterans are part of the aging population. The risk of chronic diseases rises as a population ages [4]. The combination of aging and physical impairments is associated with a functional disability in basic and instrumental activities of daily living. As a result, elderly people, especially those with handicapped, face disabilities and dependency, resulting in mental health problems and a decline in quality of life and life satisfaction [5]. To our knowledge, most of the veterans with ankle-foot injuries have rarely used orthosis or other mobility aids in their life due to the unavailability of service providers or to inappropriate devices. In this situation, mobility and transfer problems increase and limit individuals from performing routine activities at home or in the community. These individuals are underrepresented in society and are faced with restrictions in situations such as employment and educational positions.

According to the WHO definition, health-related quality of life (HRQOL) includes a person’s perception of his/her position in life within the culture and the living systems that consider their goals [6]. Various factors, including individual and environmental characteristics, have been recognized as determinants of QOL [7]. From a healthcare perspective, HRQOL is related to how a certain disorder, particularly one that is not life-threatening, affects the life of a patient. As many studies have indicated, chronic and long-term disorders lead to unexpected mental problems and a dramatic decline in HRQOL levels [8–10]. Phantom pain, low back pain, and a decreased ability to perform activities of daily living were reported as the strongest determinants of HRQOL in veterans with transfemoral amputations [8]. A negative correlation between the number of injuries and HRQOL in blind Iranian veterans has been supported [9]. Quality of life in veterans with bilateral lower limb amputations has been associated with pain and poor general health [10].

Dependence on others for the performance of daily affairs is a predictable result of such disabilities, particularly in the aging population, where many people have lost some of their previous abilities [11]. Providing appropriate services to these people can reduce their dependency and have a positive impact on increasing their quality of life. The practice of employing quality of life measures in medical research of veterans has grown in recent years [8, 12, 13]. In the current study, the authors examined the HRQOL of Iranian veterans with ankle-foot injuries resulting from the Iran-Iraq war. Evaluation of the level of dependence required to perform ADLs and IADLs determines what types of care are needed at home or in the community. As a result, we evaluated ADL and IADL abilities in a group of veterans with ankle-foot injuries in a nationwide study.

## Methods

In a cross-sectional study between 2014 and 2016, 1079 veterans with ankle-foot injuries from 11 provinces across the country were enrolled. The study group consisted of veterans who have suffered from neuromusculoskeletal problems in the ankle and/or foot due to one of the following reasons: 1) ankle and/or foot trauma and partial amputation due to a direct bullet or fragments hit, 2) leg or thigh trauma that led to neuromusculoskeletal disorders in the ankle and foot and 3) neuromusculoskeletal damage of the lower extremity leading to dysfunction of the ankle and foot that could be compensated by ankle-foot orthotic interventions. Those with an amputation at the level of the ankle or higher and central nervous system damage were excluded. Informed consent was obtained from all participants. The orthopedists examined the injured foot, and trained experts collected demographic, quality of life and ADL/IADL ability data. Demographic information included age, gender, marital status, disability percent (5% to 70%), education level (illiterate, under high school diploma, high school diploma and university education), employment (unemployed, employment status and employed), having injuries other than ankle-foot trauma, a history of hospitalization during the year preceding the study and body mass index (BMI).

“Disability percent” or “Disability rate”: According to the legislation that was passed by Parliament on June 21st, 1995, and approved by the Cabinet on December 13th, 2009, the medical commission of the Veterans and Martyrs Affair Foundation and the Armed Forces Medical Council are responsible for determining disability ratings, which form the basis for entitlement and benefits of veterans with disability. The overall disability rating is represented by a percentage (5% to 70%) and based on the level of physical and/or mental impairment [1].

“Employment status”: According to the Iranian Parliament act passed on February 14th, 1983, if certain disability criteria have been met, disabled veterans who are either employed by the government or serve on active duty in the Armed Forces and are no longer able to continue their work, are eligible to receive their full monthly salary until they retire; for others, they are entitled to receive a monthly compensation based on their education, disability rate and dependency [14].

ADL and IADL abilities were assessed by the Barthel ADL and Lawton IADL Indexes, respectively. Activities of daily living are a set of necessary activities that people tend to do routinely in life without assistance. The Barthel scale was introduced in 1965 and yields a score of 0 to 100 in reference to the performance of these activities, including eating, bathing, grooming, dressing, toilet use, bowel and bladder continence, transfer, moving and using stairs [15]. ADLs mostly occur in connection with IADLs, which refer to slightly more complex skills. IADLs include actions that are not necessarily required on a daily basis but are important for independent living. The Lawton scale was introduced in 1969 and includes the following activities: the ability to use a telephone, shopping, food preparation, housekeeping, laundry, mode of transportation, responsibility for own medications and the ability to handle finances [16]. IADL ability is scored on a 3-point scale and summed, with a range of 0 to 16. Higher scores on both scales indicate both greater function and independence.

Quality of life was measured using the 36-item Short Form Health Survey (SF-36). The SF-36 is a generic tool that can be used for both the general population and different patient groups. This questionnaire consists of 36 questions that measure 8 health-related concepts. This questionnaire also provides 2 summary scales: the physical component summary (PCS) and the mental component summary (MCS). Scores on each of the subscales range from 0 to 100, with 0 representing the worst health-related quality of life and 100 representing the best. This questionnaire has been validated in the Iranian population [17]. Data for the general Iranian population were collected from a population-based study of a random sample of 4163 individuals living in Iran. The SF-36 scores were compared between the veterans with ankle-foot injuries and the general population study [17]. In addition, HRQOL scores of bilateral lower limb amputee (BLLA) veterans were compared with the present results. The information originated from the same project in 2007 that involved 327 Iranian BLLAs [11]. To collect data, semi-structured interviews were conducted by 3 trained health workers. Each participant was interviewed face-to-face (20–30 min).

Statistical analysis was done using the Statistical Package for the Social Sciences 16.0 (SPSS 16.0). Quantitative variables are reported as the mean ± standard deviation, and qualitative variables are presented as frequencies and percentages. The relationships between the quantitative demographic characteristics and ADL/IADL and PCS/MCS scores were examined by Pearson’s correlation coefficient. To evaluate intra-correlations between the quantitative rating variables, repeated measures analysis of variance (ANOVA) was applied. The patients’ scores on the SF-36 were compared with those of bilateral lower limb amputees and of the general Iranian population using one-sample t-tests. *P* values <0.05 were considered significant. We performed multiple linear regression analyses to determine the variables that contribute most to the health-related quality of life in veterans with ankle-foot injuries. The PCS and MCS were used as dependent variables. Apart from demographic characteristics, ADL and IADL scores were considered independent variables. The variables that showed a significant *P* value were entered in the regression model.

## Results

The mean age at the time of the study was 52.11 ± 8.29 years, and the average age at the time of casualty was 22.1 ± 3.2 years. Almost all participants were male (97.5%, *n* = 1052) and married (97.6%, *n* = 1053). The education levels were mostly at the level of under high school diploma (47.0%, *n* = 508). Nearly one-fifth (18.0%, *n* = 194) of the study group had a history of hospitalization during the year preceding the study. The average BMI was 27.15 ± 7.41. Table 1 gives further details about the demographic characteristics.

**Table 1:** Demographic characteristics of veterans with ankle-foot injuries (*n* = 1079)

Item	Demographic characteristics	Frequency	Percent (%)
Age group	Less than 35 years	14	1.3
	35–44 years	96	9.0
	45–54 years	677	62.7
	55–64 years	213	19.7
	65 years and more	79	7.3
Disability percent	Less than 25%	231	21.4
	25%–49%	661	61.3
	50%–69%	137	12.7
	70%	50	4.6
Education level	Illiterate	86	8.0
	Under high school diploma	508	47.0
	High school diploma	303	28.1
	University education	182	16.9
Employment	Employed	301	27.9
	Employment status	494	45.8
	Unemployed	284	26.3
Other injuries^a^	Chemical	140	12.4
	Mental health	337	29.8
	Spinal cord	37	3.3
	Physical (head/face, trunk, upper/lower limbs)	117	10.3
	None	501	44.2
BMI	Underweight	17	1.6
	Normal weight	327	30.3
	Overweight	514	47.6
	Obese	221	20.5

*BMI* Body Mass Index

^a^The total number of other injuries is more than the sample size. Some participants had a total of more than one injury

The mean number of ADL tasks was 83.9 ± 15.9, with a minimum score of 10.0 in 0.1% (*n* = 1) and a maximum score of 100.0 in 17.7% (*n* = 191) of the participants. The highest independence level was in feeding (95.0%), while going up/down stairs (17.1%) showed the highest level of dependence (Table 2). Using stairs (50.4%), transfer (28.2%) and mobility (23.8%) were activities with the highest need for assistance. The average score of IADL tasks was 10.3 ± 3.8; from 0.1% (n = 1) who was entirely dependent with a score of 0 to 8.6% (*n* = 93) who were fully independent with a score of 16. The highest level of dependence was observed in laundry (46.0%) and housekeeping (45.2%) activities, and more than one-third of the participants needed help with transport (36.2%). In contrast, using a telephone (82.6%) and responsibility for own medications (69.0%) had the most independence.

**Table 2:** ADL and IADL abilities in veterans with ankle-foot injuries (*n* = 1079)

Activities		Independent (%)	Need Help (%)	Dependent (%)
ADLs	Feeding	1025 (95.0)	45 (4.2)	9 (0.8)
	Bathing	891 (82.6)	–	188 (17.4)
	Grooming	893 (82.8)	–	186 (17.2)
	Dressing	851 (78.9)	194 (18.0)	34 (3.1)
	Toilet use	898 (83.2)	138 (12.8)	43 (4.0)
	Bowel continence	953 (88.3)	92 (8.5)	34 (3.2)
	Bladders continence	785 (72.7)	264 (24.5)	30 (2.8)
	Transfer	736 (68.2)	304 (28.2)	39 (3.6)
	Mobility	719 (66.6)	257 (23.8)	103 (9.6)
	Using stairs	351 (32.5)	544 (50.4)	184 (17.1)
IADLs	Ability to use telephone	891 (82.6)	141 (13.1)	47 (4.3)
	Shopping	502 (46.5)	367 (34.0)	210 (19.5)
	Food preparation	386 (35.8)	306 (28.3)	387 (35.9)
	Housekeeping	342 (31.7)	249 (23.1)	488 (45.2)
	Laundry	417 (38.6)	166 (15.4)	496 (46.0)
	Mode of transportation	564 (52.3)	391 (36.2)	124 (11.5)
	Responsibility for own medications	745 (69.0)	172 (16.0)	162 (15.0)
	Ability to handle finances	668 (62.0)	294 (27.2)	117 (10.8)

*−* No data, *ADL* activity of daily living, *IADL* Instrumental activities of daily living

The means of the MCS and the PCS were 42.56 ± 21.15 (0.0–100.0) and 33.55 ± 17.82 (3.13–93.75), respectively. The mean SF-36 scores of the veterans with ankle-foot injuries were significantly lower than those of the general Iranian population and of bilateral lower limb amputee veterans on all eight measures (*P* < 0.001) (Table 3).

**Table 3:** SF-36 quality of life measure scores in veterans with ankle-foot injuries (Mean ± SD)

Measure	Veterans with ankle-foot injuries (*n* = 1079)	Bilateral lower limb amputee veterans (*n* = 327)	General Iranian population (*n* = 1997)
Physical functioning	47.21 ± 23.40	54.50 ± 24.45	85.3 ± 20.8
Role physical	25.81 ± 34.67	50.04 ± 25.05	70.0 ± 38.0
Bodily pain	27.88 ± 21.61	47.97 ± 24.02	79.4 ± 25.1
General health	33.29 ± 20.00	55.30 ± 26.71	67.5 ± 20.4
Vitality	45.33 ± 20.32	63.46 ± 23.70	65.8 ± 17.3
Social functioning	45.71 ± 26.24	66.67 ± 26.74	76.0 ± 24.4
Role emotional	30.80 ± 39.89	63.14 ± 26.77	65.6 ± 41.4
Mental health	48.39 ± 20.68	62.51 ± 25.36	67.0 ± 18.0

Comparisons were made between the data of veterans with ankle-foot injuries and those of bilateral lower limb amputee veterans, and between the data of veterans with ankle-foot injuries and those of general Iranian population; *P* < 0.05 for all the comparisons

After entering the desired variables into the regression model, significant relationships and differences were assessed among them, and the outcomes are described in Tables 4 and 5. The results obtained from the multiple linear regression analyses showed that disability rate, additional injury, ADLs and IADLs (P < 0.001) were predicting factors for poor physical health-related quality of life (Table 6). The analyses also showed that employment, additional injury, history of hospitalization, ADLs and IADLs (*P* < 0.05) were determinants of poor mental health in veterans with ankle-foot injuries.

**Table 4:** Relationship between qualitative variables and ADLs, IADLs, MCS and PCS in veterans with ankle-foot injuries

Item	ADLs		IADLs		MCS		PCS	
	r	*P*	r	*P*	r	*P*	r	*P*
Age	−0.158	<0.001	−0.204	<0.001	−0.063	0.053	−0.060	0.039
Disability rate	−0.151	<0.001	−0.041	0.018	−0.037	0.224	−0.170	<0.001
BMI	0.017	0.750	0.061	0.140	0.054	0.83	−0.010	0.750
ADL	–	–	–	–	0.352	<0.001	0.345	<0.001
IADL	–	–	–	–	0.356	<0.001	0.329	<0.001

r. Correlation coefficient, − No data, *BMI* Body mass index, *ADL* Activity of daily living, *IADL* Instrumental activities of daily living, *PCS * Physical component summary, *MCS* Mental component summary

**Table 5:** Differences in ADL, IADL, MCS and PCS between quantitative variable groups in veterans with ankle-foot injuries

Item	ADL		IADL		MCS		PCS	
	Mean ± SD	*P*	Mean ± SD	*P*	Mean ± SD	*P*	Mean ± SD	*P*
Education								
Illiterate	75.46 ± 22.17	<0.001	7.1 ± 3.95	<0.001	35.96 ± 17.32	0.006	29.07 ± 14.20	0.252
Under high school	83.71 ± 15.36		10.12 ± 3.76		41.13 ± 19.24		33.35 ± 16.93	
High school	84.71 ± 15.28		11.01 ± 3.50		44.87 ± 23.26		35.06 ± 19.36	
University education	87.28 ± 13.85		11.21 ± 3.23		46.06 ± 23.37		33.81 ± 18.97	
Employment								
Employed	80.15 ± 17.78	<0.001	9.38 ± 4.01	<0.001	35.77 ± 18.84	<0.001	31.83 ± 16.88	0.001
Employment status	88.44 ± 13.44		11.48 ± 3.38		48.18 ± 23.63		37.20 ± 19.32	
Unemployed	82.57 ± 16.26		9.93 ± 3.77		41.29 ± 19.74		32.19 ± 17.04	
Additional injuries								
Yes	82.56 ± 16.64	0.011	10.24 ± 3.86	0.715	39.61 ± 20.72	<0.001	30.81 ± 16.14	<0.001
No	85.16 ± 15.26		10.36 ± 3.69		45.23 ± 21.19		36.03 ± 18.89	
Hospitalization								
Yes	77.55 ± 19.57	<0.001	9.16 ± 4.02	<0.001	35.47 ± 19.38	<0.001	28.03 ± 15.53	<0.001
No	85.32 ± 14.72		10.55 ± 3.67		44.11 ± 21.21		34.75 ± 18.07	

*ADL* Activity of daily living, *IADL* Instrumental activities of daily living, *PCS * Physical component summary, *MCS* Mental component summary

**Table 6:** Determinants of the physical and mental component summaries of the quality of life in veterans with ankle-foot injuries

Determinants	B	SE	t	*P*
PCS				
Constant	4.902	3.513	1.395	0.163
Age	0.045	0.063	0.707	0.480
Disability rate	**−**0.128	0.035	−3.607	<0.001
Employment	2.210	1.772	1.247	0.213
Additional injuries	3.716	1.033	3.598	<0.001
Hospitalization	2.538	1.352	1.877	<0.061
ADLs	0.195	0.042	4.684	<0.001
IADLs	0.929	0.176	5.262	<0.001
MCS				
Constant	−7.991	4.374	−1.827	0.065
Education	1.453	2.668	0.545	0.586
Employment	7.037	2.108	3.338	0.001
Additional injuries	4.758	1.196	3.979	<0.001
Hospitalization	4.215	1.565	2.694	0.007
ADLs	0.235	0.049	4.836	<0.001
IADLs	1.140	0.209	5.453	<0.001

*B* Regression coefficient, *SE* Standard error, *ADL* Activity of daily living, *IADL* Instrumental activities of daily living, *PCS * Physical component summary, *MCS* Mental component summary

## Discussion

The current data described that quality of life of veterans with ankle-foot disorders were below the normal Iranian population QOL scores in each dimension [17]. The same results were obtained in comparison with the quality of life scores of veterans with bilateral lower limb amputation [11]. Physical pain were determined to be the main complications, whereas mental health and physical functioning were shown to be the most intact among all other aspects. However, the significant differences in each area of QOL between our study population and the comparison groups indicated that veterans with ankle-foot injuries were experiencing a serious condition in terms of quality of life. In a study of male Iranian veterans who had been living with unilateral lower extremity amputations for an average of two decades, role physical achieved the highest scores, while physical functioning had the lowest [8], which contradicted our results. Similar to the present study, the mean MCS scores were higher than those of the PCS among the aforementioned group [8]. Comparing QOL scores between two groups of patients with ankle and hip arthrosis in a cohort study showed that the SF-36 subscale scores in both groups were below those of the normal population of Canada. In addition, the mental and physical problems associated with ankle arthrosis are as severe as those associated with hip arthrosis [18]. Patients who have undergone arthrodesis for ankle arthritis were studied after twenty years, and their QOL in terms of physical function, emotional disturbance and bodily pain showed significant differences from the normal population [19]. A negative impact of chronic plantar heel pain on HRQOL has been confirmed, apart from the influences of age, sex and body mass index [20]. Among individuals affected by rheumatoid arthritis, those with mild disease expression in the foot have demonstrated a dramatic difference in quality of life that showed a statistically significant relationship with age [21]. By comparing QOL scores between diabetic patients with and without foot ulcers, significant differences were found in physical functioning, social functioning and general health [22].

On the other hand, ADL/IADL scores of individuals with ankle-foot injuries showed that movement, transportation and using stairs were the most frequently problematic of the daily activities that required the help of others. Additionally, laundry was found to be the activity for which more than four-fifths of veterans with ankle-foot injuries were partially or completely dependent. Feeding, telephone use and taking responsibility of medicine were activities with the highest level of independence. Similar studies have shown that lower limb amputees needed help in transfer more than other daily activities, but food preparation and housekeeping were the most frequent causes of dependency in IADL [9, 10]. Limitations in activities of daily living and mobility were reported in half of the population with long-term lower extremity injuries [11]. Additionally, the verified predictors of functional ability in older people were foot and ankle characteristics, particularly ankle flexibility, plantar tactile sensation, and strength of toe plantar flexor muscles [12].

A greater disability percent, as well as having injuries other than ankle-foot, correlated with ADL/IADL limitations. All these factors had a predicting role in the PCS, while the latter three were determinants of the MCS. Most of the participants in this study had been suffering from two or more injuries, which increased their dependence on others. According to our results, higher QOL scores would be created by higher independence. Some previous studies also showed that long-term disabilities, particularly those affecting routine daily activities, have an indirect correlation with mental health and QOL [7–10]. The present results are also similar to those of some Iranian studies of similar groups; more physical injuries was a factor in the decreasing ability of personal-social actions [23–27]. The association between QOL and additional injuries was confirmed in previous studies on Iranian veterans suffering from physical injuries including extremity, trunk, head and face injuries, as well as from mental disorders, and also who were blind or exposed to sulfur mustard [12, 28].

Since the majority of cases involved individuals between 45 to 54 years old, it was expected that they were employed. However, a quarter of them were unemployed. Almost all of them were male and married, so having a job was necessary to meet the cost of living. Employment and its subsequent presence in the community have been shown to increase QOL in those with lower limb injuries [29]. Additionally, it has been shown that employment of people with lower extremity injuries is dependent on the type of job, mobility level and the use of comfortable mobility aids [30].

Hospitalization during the year preceding the study was related to both the MCS and the PCS. It could be concluded that hospitalization might be a consequence of additional injuries, which are significant factors for increased disability. Since the relationship between disability and QOL has been indicated in previous studies, the association between hospitalization and QOL is probable [31].

The education level of more than half of our veterans was at the level of under a high school diploma. Studies of unilateral and bilateral blind, lower and upper limb amputated and chemically injured Iranian veterans of the same age showed that veterans with ankle-foot injuries were 10 to 30% less educated [23, 24, 32, 33]. Difficulties in commuting to school due to said injuries are among the possible causes for this result. The second plausible reason is the lack of allocation of special education services for veterans who have only suffered ankle-foot injuries compared with those with greater disabilities. Previous studies have shown that veterans with a higher disability percent are more educated; therefore, the second reason seems more credible [23, 26, 32, 33].

Older individuals with higher disability rates obtained lower scores in both indexes of daily activity abilities. A study of Iranian veterans found that the aging period in this group begins an average of a decade earlier than the normal population [27]. With the mean age of 52 in the current study, the majority of the participants had just started aging or were already elderly. Approximately 95% of the normal Iranian population that are one decade older (at the beginning of elderly age) were independent in ADLs [34]. In this regard, approximately one-fifth of our group was entirely independent in ADLs/IADLs, while the rest of them needed partial or complete help probably due to long-term physical impairments.

For the first time, quality of life and the ability to perform ADL and IADL in veterans with ankle-foot disorders were assessed. This study showed that HRQOL scores were significantly different between those who were able and those who were unable to perform ADLs/IADLs. In this regard, prescribing and manufacturing appropriate orthotics and regular examination can be effective in boosting the ability of these individuals to perform daily activities. Although the study was conducted in the center of each province, the inability to recruit all the study population in each area was the major limitation in this survey. Many were from rural areas, and due to the matter of distance they did not gain the opportunity to participate in this study. In addition, some addresses and phone numbers had been changed, and we did not have access to some of them. Another limitation of the study was using different medical teams in each province, which possibly reduced the validity of the study. HRQOL scores in veterans with ankle-foot injuries were dramatically lower than not only those of the normal population but also those of BLLAs with 70% disability rates (except in partial amputations). As a result, future research focusing on the causes of this difference seems necessary. Future studies with orthotic interventions and comprehensive rehabilitation services are recommended particularly since many of the veterans were at the beginning of the aging period.

## Conclusion

These results illustrate that the QOL of veterans with ankle-foot injuries was significantly lower than that of the normal Iranian population and of BLLAs in all the investigated dimensions. The lowest score was observed in the bodily pain scale, while the highest score reported for mental health. Mobility, transportation and using stairs were the most frequent problems in their daily activities. The highest ability was observed in the activities of eating and telephone use in ADLs and IADLs, respectively. The main determinants for the PCS were a higher disability rate, having additional injuries, and limited ADLs and IADLs. The major predicting factors for the MCS were unemployment, suffering additional injuries, a history of hospitalization, and a higher dependency in ADLs and IADLs.

## Abbreviations

ADL: Activity of daily living
BLLAs: Bilateral lower limb amputees
BMI: Body mass index
HRQOL: Health-related quality of life
IADL: Instrumental activity of daily living
MCS: Mental component summary
PCS: Physical component summary
